# Pedagogical impact of different levels of e-learning teaching during anesthesia residency: a randomized clinical trial

**DOI:** 10.1186/s12909-026-09225-4

**Published:** 2026-04-29

**Authors:** Jean Selim, Juliette Thill, Julien Burey, Emmanuel Besnier, Maud Lambert, Antoine Lefevre-Scelles, Julien Abily, Goia Gastaldi, Etienne Allard, Jean-Luc Hanouz, Benoît Veber, Bertrand Dureuil, Caroline Thill, Vincent Compère

**Affiliations:** 1 Department of Anesthesiology and Critical Care, Rouen University Hospital, 1 Rue de Germont, Rouen, 76000 France; 2 Univ Rouen Normandie, INSERM EnVI UMR 1096, Rouen, 76000 France; 3 Department of Anesthesiology, Le Havre Hospital Center, Avenue Mendès France, Montivilliers, 76290 France; 4 Department of Anesthesia and Intensive Care, Caen Normandie University Hospital, Caen, 14000 France; 5 Department of Biostatistics, Rouen University Hospital, Rouen, France; 6 Normandie Univ, UNIVROUEN, INSERM U982, Rouen, F76000 France

**Keywords:** E-learning, Anesthesia residency, Medical education, Computer-assisted learning, Clinical knowledge assessment

## Abstract

**Introduction:**

E-learning is in expansion although its pedagogical performance has not been validated in the anesthesia residency. E-learning can be divided into passive e-learning; guided e-learning, interactive e-learning, and adaptive e-learning. The objective was to compare the impact of passive e-learning versus interactive e-learning on anesthesia residents' acquisition of theoretical knowledge and clinical reasoning.

**Material and methods:**

Anesthesia residents were randomized into a Passive E-learning group (Pass-EL group) and an Interactive E-learning group (Int-EL group). The Pass-EL group had documents sent by email and the Int-EL group had access to a pedagogical internet platform. A test composed of one clinical case, evaluating the clinical knowledge, and, 15 script concordance tests (SCT), evaluating the clinical reasoning, was conducted before and after e-learning. The outcome of the comparison was the means of the paired within-subject differences (delta) of clinical knowledge scores (pre-and post-test) between the two groups.

**Results:**

Fifty-four residents were randomized in this prospective multicentric study in Normandy, France. Twenty-eight in the Int-EL group and 26 in the Pass-EL group. The mean of the paired within-subject differences of theoretical clinical knowledge scores after *versus* before e-learning was significantly higher in the Int-EL group compared to the Pass-EL group (Int-EL group 8.5 [-1.0; 19.5] vs. Pass-EL group 5.4[-6.0; 21.5], mean delta score difference: 3.1 [0.1; 6.1], *P =* 0.045). There was no difference for SCT. Seven residents (25%) reached out to the mentor within the Int-EL group.

**Conclusion:**

Interactive e-learning showed efficacy for theoretical clinical knowledge but did not improve clinical reasoning.

**Trial registration:**

www.clinicaltrialsgov NCT07121400, registered 2025–08-13.

**Supplementary Information:**

The online version contains supplementary material available at 10.1186/s12909-026-09225-4.

## Introduction

During residency, learners must complete a high level of theoretical clinical knowledge and clinical reasoning. Classroom instruction remains the most traditional and common form of medical education but can limit the acquisition of knowledge (passive learning, one-size-fits-all approach, limited critical thinking and application, and reduced collaboration and interaction) [1]. With the development of the Internet, learners have access to a wide range of courses and are less and less interested in traditional classes. Thus, the traditional pedagogical system is changing to include new active pedagogical strategies focused on the student [2]. In the context of medical education, Electronic-learning (E-learning) has been developed and can be defined by Sangrà V et al.: “*E-learning*, *is an approach to teaching and learning, representing all or part of the educational model applied, that is based on the use of electronic media and devices as tools for improving access to training, communication, and interaction and that facilitates the adoption of new knowledge, skills and/or behavior/attitude”* [3].

E-learning is associated with several advantages in medical education. First, it does not depend on a teaching place or the teacher's time. Second, it can also provide active and personalized teaching of the student, according to the level of e-learning [4]. Also, the need for e-learning is now inseparable from any medical training, particularly with the adoption of competency-based medical education. Indeed, e-learning offers numerous advantages: it provides accessibility and flexibility for learners, allows for the integration of modern technologies, ensures scalability, supports the globalization of education, adapts to rapid changes, leverages enhanced data analytics and is cost-effective. Recent studies and meta-analyses showed that e-learning had a positive impact on theoretical acquisition, medical reasoning, and, technical skills even if they do not show any superiority compared to classical teaching [5–8]. This can be explained by the e-learning courses’ heterogeneity. In anesthesiology, few studies have evaluated the impact of e-learning on residents and the results are controversial. Thus, Chu et al*.*highlighted the value of a 10-month e-learning program to prepare for the beginning of the anesthesiology residency at Stanford University [9]. In contrast, a randomized controlled trial by Hards et al*.*showed no difference in cardiac arrest management between e-learning and traditional teaching training [10]. The different results observed between studies can be explained by the existence of various levels of e-learning. E-learning in medical education can be stratified into four levels: passive, guided, interactive, and adaptive. Passive e-learning offers static content with limited engagement, making it cost-effective but less interactive. Guided e-learning provides structured pathways with interactive elements, fostering more engagement but with less flexibility. Interactive e-learning actively involves learners in simulations and decision-making, enhancing critical thinking, though it requires more resources. Adaptive e-learning personalizes the learning experience by adjusting content to individual needs and optimizing outcomes, but it is technologically complex and costly. Each level has its advantages and disadvantages, and the choice depends on the educational objectives and available resources [2].

The objective of this study was to compare the impact of e-learning on theoretical knowledge and clinical reasoning between a group of anesthesia residents who received Passive-E-learning (Pass-EL group) and a group who received Interactive-E-learning (Int-EL group).

## Material and methods

### Residents’ selection

This prospective multicenter, randomized, controlled study was conducted from February to June 2016. All anesthesia residents from the first to the fourth year were included. Exclusion criteria included residents missing the pre-or post-tests or refusing to participate in the training program. The Ethics and Evaluation Committee for Non-Interventional Research approved the study (Ethics Committee N° E2015-53) and waived the requirement for written informed consent for each resident for this study according to the current French law [11]. This study was constructed using a validated Medical Education Research Study Quality Instrument (MERSQI) (Supplementary File 1) [12]. Trial registration: www.clinicaltrialsgov NCT07121400, registered 2025–08–13.

### Study procedures

Residents first have a pre-test consisting of a clinical case and a script concordance test (SCT). The clinical case was designed to evaluate theoretical clinical knowledge. The clinical case scored 50 points and consisted of 4 open-ended questions (12,5 points for each question) on the management and complications of the diabetic patient in anesthesia (preoperative anesthetic evaluation, treatment management, anesthesia management, postoperative complications). The management of diabetes was selected as the focus due to its significance as a common comorbidity and the observed gap in residents' structured understanding of its implications in anesthesia.

The SCT was designed to evaluate clinical reasoning [13, 14]. The SCT consisted of 15 questions on the management of the diabetic patient in anesthesia (degenerative neuropathy, ischemic cardiopathy, intubation, management of hyperglycemia, postoperative complications) and was also scored out of 50 points. Residents had 15 min for the clinical case and 15 min for the SCT. Students were automatically timed out when the time limit was reached. For each SCT, the resident had the choice between 5 rated responses ranging from −2 to + 2 (−2, the hypothesis is very unlikely; −1, the hypothesis is less likely; 0, the hypothesis is equally likely; + 1, the hypothesis is more likely; + 2, the hypothesis is very likely) (Supplementary File 2).

The pre-test was conducted in a double-blind way, each resident did not know in which group they would be. Each test had a double-blind correction by two senior anesthesiologists. Residents were then randomized to either the Int-EL group or a Pass-EL group. A computer-generated randomization sequence (1:1: allocation ratio) was used to assign residents to one of the two e-learning groups. The e-learning materials were emailed to each resident participating in the study by an independent anesthesiologist from the department. The Int-EL group had full access to a pedagogical internet platform with online documents; national experts’ recommendations on the management of diabetic patients in anesthesia, slide presentation lectures on the topic by local university teachers, and, the possibility to contact a virtual mentor (anaesthesiologist of our department) by email for interactive discussion or questions about the topic [15–17]. The platform was divided into 3 learning modules: preoperative, intraoperative, and postoperative management of diabetic patients. These learning modules were developed by two faculty members from the Department of Anesthesiology and Critical Care, both qualified in medical education. The conception of this e-learning platform was realized following a very precise pedagogical pathway: Analysis, Design, Development, Implementation, and Evaluation (ADDIE model) of the pedagogical project [18–20]. To access new documents the resident had to progress from module 1 to module 2, and then to module 3. To progress to the next module, the resident had to validate at least a series of 4 multiple-choice questions (MCQs) on 5. If the score was under 4 out of 5, the student was encouraged to study on the pedagogical platform again before attempting the assessment. Once the module was completed, students had the option to retake the multiple-choice questions and revisit the educational materials from the previous section if they wished. In addition to the multiple-choice questions and optional virtual mentor, no other interactive elements were incorporated. The e-learning platform was hosted on a private server and accessed online. Educational files were uploaded via FTP, and the user interface was developed using JavaScript, HTML5, and CSS3. MySQL managed the database, with user data secured through encryption. Participants accessed personalized environments using a session-based login system. The platform was created with design principles such as ergonomics, adaptability for students, efficiency, and accessibility integrated.

The Pass-EL group only received by email the documents (French experts’ recommendations and slide presentation lectures on the management of diabetic patients in anesthesia) and did not have access to the pedagogical internet platform.

The e-learning training period was one month for both groups. We then conducted, in both groups, a post-test composed of the same clinical case and the same SCT. The time allotted for the post-test was identical to that of the pre-test, and students were not allowed to access external resources during either test. Each test had a double-blind grading by two senior anesthesiologists. At the end of this examination, a survey evaluating the teaching tool was sent to all residents. The tests were developed by two experts in anesthesiology and medical education within our department. The residents in the Pass-EL group had the option to access the platform at the end of the study to complete the e-learning course, should they choose to do so.

### Outcome measures

The primary outcome was the comparison of the theoretical clinical knowledge score between the two e-learning groups (as measured by clinical case tests). The score used was the means of the paired within-subject differences (delta) of clinical knowledge scores (pre-and post-test).

The secondary outcomes were:

- The comparison of clinical reasoning scores between the two e-learning groups was evaluated by the paired within-subject differences (delta) SCT scores (pre-and post-test).
- Self-reported difficulty (“easy”, “medium to difficult” and “difficult”) in accessing the pedagogical platform for the Int-EL group and the access to the documents for the Pass-EL group.
- Self-reported difficulty (“easy”, “medium to difficult” and “difficult”) to validate the MCQs felt by the students of the Int-EL group.
- Self-reported e-learning usage between the two e-learning groups of the means.
- Student satisfaction score between the two e-learning groups using a scale of 0 to 10.

### Statistical analysis

As this work was a pilot study with no data available in the literature, no calculation of the number of students was performed. A sample size of 20 students per group was retained. Statistical analyses were performed with the Statistical Software SAS (version 9.4, SAS Institute, Cary, NC). A two-sample Student’s t-test was used to compare, between randomization groups, the means of paired within-subject differences of scores after *versus* before e-learning.

To assess the validity of the total score as an indicator of resident knowledge, Pearson's correlation coefficient was calculated between the clinical case score and the SCT score on pre-test and post-test (Supplementary File 3).

This univariate analysis was complemented by an adjusted comparison using a multiple linear regression model as a sensitivity analysis to strengthen the results. This model accounted for variables such as the initial score and the student’s experience. Only the variables associated with the outcome (difference in scores between the February and June evaluations) at the chosen threshold (p ≤ 0.05) were retained in the model. The same approach used for the univariate and multivariate analyses of the primary outcome was applied to the secondary outcomes.

Continuous data were presented as mean ± standard deviation (SD) and the 95% confidence interval was estimated for the mean score differences of clinical case evaluation and SCT. Qualitative data were presented as the number of cases (n), and percentage (%). All p values were two-tailed, with statistical significance indicated by a value of P < 0.05.

## Results

**Fig. 1:**
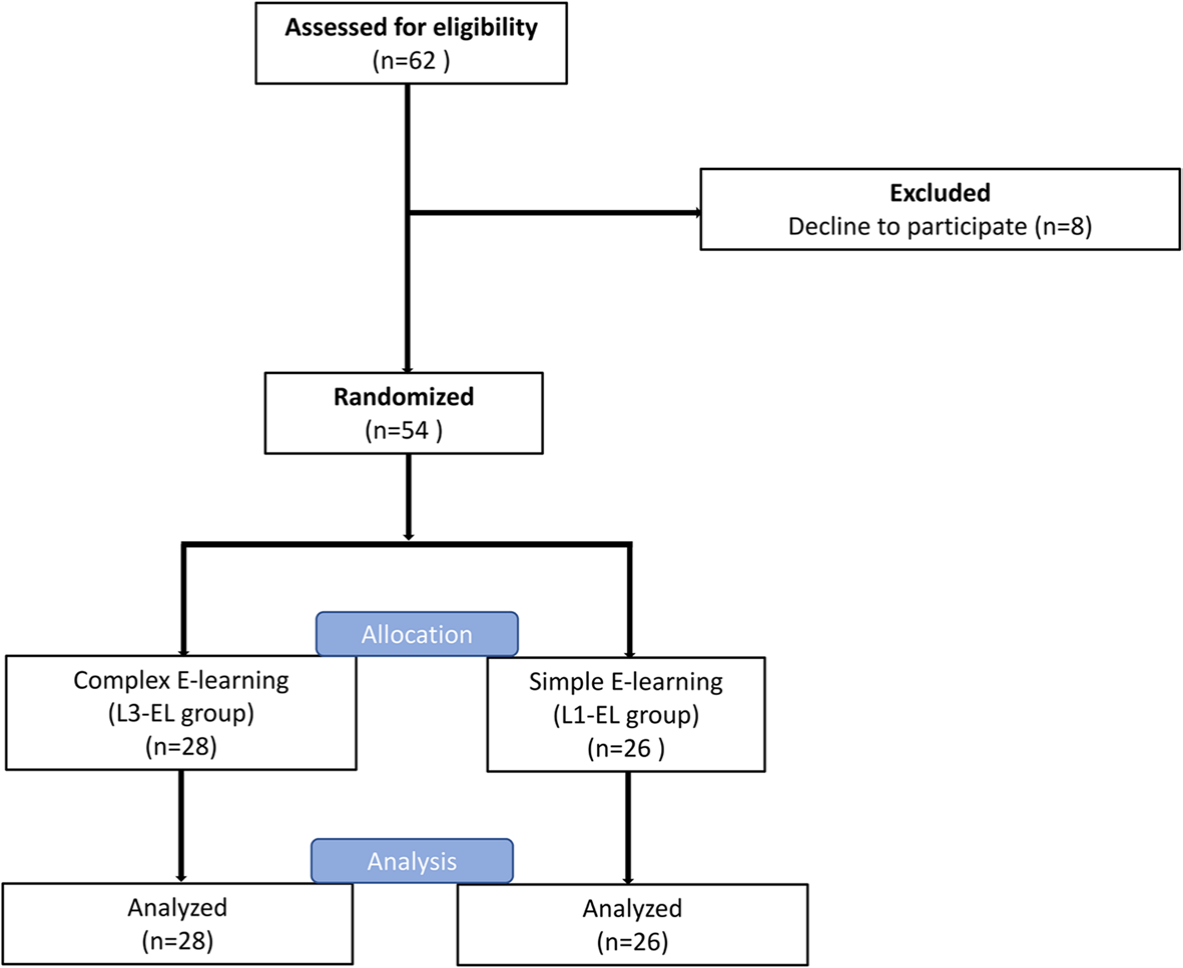
Flow chart

Sixty-two residents were assessed for eligibility. Eight were excluded because they declined to participate. Fifty-four residents were randomized with 28 in the Int-EL group and 26 in the Pass-EL group. No residents were lost to follow up in any of the groups. In the final, fifty-four residents were analyzed (Fig. 1). Genre distribution, post-graduate year distribution, national rank exam, and, pre-test performance of the residents are represented in Table 1.

**Table 1:** Population characteristics

	Global population (*n* = 54) (%)	**Passive e-learning** Pass-EL group (*n =* 26) (%)	**Interactive e-learning** Int-EL group (*n =* 28) (%)
Gender			
Male	33 (61)	17 (65)	16 (57)
Female	21 (39)	9 (35)	12 (43)
Post-graduate year			
First	19 (35)	9 (35)	10 (36)
Second	11 (20)	6 (23)	5 (18)
Third	10 (19)	6 (23)	4 (14)
Fourth	14 (26)	5 (19)	9 (32)
University Hospital			
- Rouen	43 (80)	20 (77)	23 (82)
- Caen	11 (20)	6 (23)	5 (18)
National rank exam before anesthesia residency			
(ranking out of 8 000 interns)	2821 ± 800	2889 ± 836	2745 ± 770
Pre-test performance			
Clinical case (points)	20.3 ± 4.78	20.4 ± 5.3	20.4 ± 4.3
SCT (points)	30.5 ± 5.2	30.8 ± 5.0	30.4 ± 5.4
Post-test performance			
Clinical case (points)	27.3 ± 6.89	25.8 ± 7.12	28.75 ± 6.49
SCT (points)	33 ± 5.36	33.8 ± 4.43	32.1 ± 6

The clinical case and the SCT were scored 50 points each

For continuous measurements, data are expressed as mean ± standard deviation. For qualitative parameters, data are presented as n (% of patients)

*SCT *Script concordance test, *Pass-EL group *Level 1-E-learning group, *Int-EL group *Level 3-E-learning group

### Primary outcome: clinical knowledge

The mean of the paired within-subject differences of theoretical clinical knowledge scores after *versus* before e-learning was significantly higher in the Int-EL group compared to the Pass-EL group (Int-EL group 8.5 [−1.0; 19.5] vs. Pass-EL group 5.4 [−6.0; 21.5], mean delta score difference: 3.1 [0.1; 6.1], *P =* 0.045) (Table 2). The sensitivity analysis, adjusted for the initial score and the student's experience, confirmed that the difference in theoretical clinical knowledge scores after versus before e-learning was significantly greater in the Int-EL group compared to the Pass-EL group (mean delta score difference: 3.1 [0.4; 5.7], *P =* 0.023).

**Table 2:** Mean delta scores between the Simple E-learning group and the Complex E-learning group

	**Passive e-learning** (Pass-EL group) (*n =* 26)	**Interactive e-learning** (Int-EL group) (*n =* 28)	**Mean delta score difference**	***p*-value**
Mean of the paired within-subject differences of theoretical clinical knowledge scores after *versus* before E-learning	5.4 [−6.0; 21.5]	8.5 [−1.0; 19.5]	3.1 [0.1; 6.1]	0.045
Mean of the paired within-subject differences of SCT scores after *versus* before E-learning	3.1 [−9.8; 16.6]	1.8 [−9.0; 16.6]	−1.3 [−4.3; 1.8]	0.41

For continuous measurements, data are expressed as mean [95%CI]

*SCT *script concordance test, *CI* confidence interval, *Pass-EL group *Passive-E-learning group, *Int-EL group *Interactive-E-learning group

### Secondary outcomes: clinical reasoning

There was no difference between the two groups for the means of paired within-subject differences of scores after *versus* before e-learning for the SCT (Table 2).

In the Int-EL group, the platform access was considered “easy” for 14 residents (50%), "medium to difficult" for 10 residents (35%), and, “difficult” for 4 residents (15%). Seven residents (25%) reached out to the mentor within the Int-EL group. In the Pass-EL group, document access (sent by email) was considered “easy” for 26 residents (100%). In the Int-EL group, 7 residents (25%) had considered the validation of MCQs as “difficult”. The other 21 residents In the Int-EL group did not answer this question. All the residents in the Pass-EL group confirmed that they received the documents sent by email.

The reporting time of e-learning usage (Int-EL group, 122.5 ± 91.9 min vs. Pass-EL group, 98.3 ± 66.4 min, *P =* 0.314) and the mean resident satisfaction score (Int-EL group, 5.9 ± 2.5 vs. Pass-EL group, 5.9 ± 2.2, *P =* 0.971) were no different between the two groups (Table 3).

**Table 3:** Comparison of working time and satisfaction score between the two groups

	**Global population** (*n =* 44)	**Passive e-learning** Pass-EL group (*n =* 20) (%)	**Interactive e-learning** (Int-EL group) (*n =* 24) (%)	***p*-value**
Working time (minutes)	111.5 ± 81.3	98.3 ± 66.4	122.5 ± 91.9	0.314
Satisfaction score (scale of 0 to 10)	5.9 ± 2.3	5.9 ± 2.2	5.8 ± 2.5	0.971

Data are expressed as mean ± standard deviation

*Pass-EL group* Passive-E-learning group, *Int-EL group* Interactive-E-learning group

## Discussion

### Medical evaluation and reasoning

In this dual center, randomized, controlled study, Interactive e-learning (represented by the Int-EL group) was more effective in improving theoretical clinical knowledge on the perioperative management of diabetics compared to Passive e-learning (represented by the Pass-EL group). However, we did not find any impact of the Complex e-learning training on clinical reasoning. Teaching on the pedagogical platform did not significantly increase the working time and therefore suggests an optimization of the residents' working method. However, it is important to note that this time is self-reported and therefore subject to various biases that could be influenced by the design or implementation of the new educational program. Both groups improved their performance on the post-tests for theoretical clinical knowledge with a significantly higher progression for residents in the complex e-learning group (+ 8.5 points out of 50) compared to the residents of the simple E-learning (+ 5.4 points out of 50). This significant difference of 3.1 points, equivalent to 6.2%, is noteworthy and aligns with findings from a similar study conducted on medical students focusing on hematology training [21]. However, recent meta-analyses did not find any difference in performance for knowledge and non-technical skills between e-learning and traditional learning [5, 7, 22]. The heterogeneity of e-learning programs makes probably the interpretation of pedagogical effects complex. The choice of theoretical clinical knowledge as the primary outcome may raise questions, but it seemed more appropriate for students compared to SCTs, which assess complex reasoning and are therefore more often intended for senior physicians.

The evaluation by MCQs after each module on the platform is similar to a continuous assessment mode, encouraging learners to work regularly [23]. Each module of e-learning allows the student to manage the acquisition of information promoting the acquisition of transversal skills such as autonomy and self-determination [24]. Moreover, e-learning can be used alone or in combination with classical teaching (blended learning). Several studies have shown interesting results in favor of blended learning compared to traditional teaching alone [25, 26]. We did not observe any significant difference in the declared working time between the two groups, this suggests an optimization of learning time with Complex e-learning. Furthermore, this result is consistent with a study conducted on hematology residents who showed a positive impact of e-learning on theoretical clinical knowledge about leukemia without a significant difference in working time compared to classical training [21]. The SCT is valuable for assessing decision-making in complex, real-world clinical scenarios. We use it in our study to evaluate how participants apply clinical knowledge in practical situations, crucial for improving diagnostic and therapeutic decision-making. However, we did not show any impact of e-learning on the improvement of clinical reasoning, which has been evaluated by the SCT [27]. This result is not surprising because the pedagogical platform documents offered access to theoretical knowledge and MCQ evaluations did not require any particular medical reasoning. This aspect could be improved by setting up blended learning in the form of face-to-face teaching by practice exchange groups [13, 28].

### Satisfaction

Although providing an attractive pedagogical program, the satisfaction mean score for the Complex e-learning was only 5.8 on a scale from 0 to 10. E-learning remains an interesting pedagogical tool but cannot satisfy all residents. This is in contradiction with the literature which generally shows a significant interest of students in pedagogical platforms [9]. This can be explained by the interface of our platform, which was “medium to difficult” to use for 35% of the residents and “difficult” for 15% of the residents. It is also important to note that, in addition to satisfaction and perceived effectiveness, other factors influence online learning. For example, we did not incorporate mechanisms for personalization and adaptability at the individual student level in our platform. All these factors should be considered when designing an e-learning platform to assess its relevance and potential impact.

### Limits

Although our study was prospective and randomized with a high-quality MERSQI score (15/18), it has several limitations. First of all, even if we found a significant difference between the two groups for our main result, the precision of the mean score remains low (between 0.1 and 6). This can be explained by the lack of power because of the small number of participants. Second, there is a center effect because of the limited participation of residents from the Caen University Hospital. Third, only the residents who accepted to participate in this pedagogical study were evaluated. We can think that they were more interested in new pedagogical tools than those who refused. Fourth, the fact that the pre-test and post-test are identical may also introduce a memory bias, leading to an artificial improvement in scores, a learning bias not related to the e-learning intervention, or a reduction in response variability. Fifth, the working time is self-reported, making it susceptible to various biases, which may be influenced by the design or implementation of the new educational program. Sixth, only 7 residents (25%) in the interactive e-learning group reached out to the virtual mentor, despite this being a key feature of our e-learning program. Finally, we did not assess long-term retention by the students, nor the clinical impact on patients.

## Conclusion

Complex e-learning for the management of diabetic patients in the perioperative period significantly improves the theoretical clinical knowledge of anesthesia residents compared to Simple e-learning. However, e-learning alone does not improve clinical reasoning.

## Supplementary Information

Supplementary Material 1.

Supplementary Material 2. Clinical case.

Supplementary Material 3. Pearson’s correlation analysis.

## Data Availability

The data underlying this article will be shared on reasonable request to the corresponding author.
