# Supplementary material for: Pedagogical impact of different levels of e-learning teaching during anesthesia residency: a randomized clinical trial
Source: BMC Med Educ. 2026 Apr 29;26:971. doi: 10.1186/s12909-026-09225-4 (PMC13267392; doi:10.1186/s12909-026-09225-4)
Supplement: Supplementary file 2 — Supplementary Material 2. Clinical case. [file 12909_2026_9225_MOESM2_ESM.docx]

**Supplementary File 2**

**Clinical case**

*You are seeing a 65-year-old female patient with a BMI of 28 for a low vaginal hysterectomy.*

*Medical history:*

- *Type 2 diabetes for 10 years, insulin-dependent for 1 year*
- *One stroke with no sequelae*
- *Moderate hypertension*

*Lip test: negative; Mallampati: 1; mouth opening: 40 mm; thyromental distance: 80 mm.*

*Treatment:*

*Acetylsalicylic acid*

*ACE inhibitor,*

*Metformin*

*Sulfonamide*

*Long-acting insulin (8-0-8 units)*

**Questions**

1. How do you complete the preoperative assessment?"
2. How do you manage her antidiabetic treatment before, during, and after the procedure?
3. What are the different elements you monitor during the procedure, and what measures do you implement about diabetes?

*Intraoperatively: significant bleeding (1000 ml) and conversion to laparotomy*

*Postoperatively: difficult refeeding due to severe pain and nausea. All antidiabetic medications have been discontinued.*

*Postoperative prescription: 2500 ml of 5% dextrose over 24 hours + 3 g/L NaCl + 1 g/L KCl, and intravenous Paracetamol, Ketoprofen, Tramadol, and Ondansetron.*

*On postoperative day 2: You are called to the ward because she presents with hypothermia and a urine analysis showing ++ Nitrites, ++ Leukocytes, ++ Glucose, and ++ Ketones*

1. What is your diagnosis based on this presentation? What are the possible etiologies in this context? What management do you initiate promptly?

**Script concordance tests**

| Proposed approach | But you learn that | The proposed approach becomes. |
| --- | --- | --- |
| Diagnostic option | New clinical information | -2 +2 0 +1 +2 |

(-2, the hypothesis is very unlikely; -1, the hypothesis is less likely; 0, the hypothesis is equally likely; +1, the hypothesis is more likely; +2, the hypothesis is very likely)

***You are seeing in anesthesia consultation a 65-year-old man, an insulin-dependent type 2 diabetic, for a scheduled peripheral vascular bypass.***

|  | **You are considering** | **But you learn that** | **The proposed approach becomes** |
| --- | --- | --- | --- |
| 1 | Gastroparesis. | An episode of orthostatic hypotension one month ago | -2 -1 0 +1 +2 |
| 2 | Difficult intubation | Progression of diabetes over the past 12 years | -2 -1 0 +1 +2 |
| 3 | Difficult intubation | Poorly managed diabetes, HbA1c > 8% | -2 -1 0 +1 +2 |

***This patient does not exhibit clinical angina***

|  | **You are considering** | **But you learn that** | **The proposed approach becomes** |
| --- | --- | --- | --- |
| 4 | Silent myocardial ischemia | History of stroke | -2 -1 0 +1 +2 |
| 5 | silent myocardial ischemia | Exercise capacity > 4 METs | -2 -1 0 +1 +2 |
| 6 | Silent myocardial ischemia | Poorly managed diabetes, HbA1c > 8% | -2 -1 0 +1 +2 |

|  | **You are considering** | **But you learn that** | **The proposed approach becomes** |
| --- | --- | --- | --- |
| 7 | Myocardial ischemia test | Renal impairment with proteinuria | -2 -1 0 +1 +2 |
| 8 | Myocardial ischemia test | Exercise capacity not assessable | -2 -1 0 +1 +2 |
| 9 | An echocardiogram | Two-stage exercise dyspnea | -2 -1 0 +1 +2 |

***You administered general anesthesia to this patient, who is normally treated with biguanides and long-acting insulin, for a left femoropopliteal bypass***

|  | **You are considering** | **But you learn that** | **The proposed approach becomes** |
| --- | --- | --- | --- |
| 10 | Maintain a 5% dextrose infusion at 100 ml/h throughout the procedure | A capillary blood glucose level of 11.5 mmol/L intraoperatively | -2 -1 0 +1 +2 |
| 11 | An intravenous insulin therapy | End of the procedure in 10 minutes. | -2 -1 0 +1 +2 |

***In the post-anesthesia care unit (PACU), after 30 minutes of sedation cessation, the patient shows no signs of awakening***

|  | **You are considering** | **But you learn that** | **The proposed approach becomes** |
| --- | --- | --- | --- |
| 12 | Hypoglycemia. | A continuous infusion of 5% dextrose at 100 ml/h was maintained throughout the procedure | -2 -1 0 +1 +2 |
| 13 | A hyperosmolar coma | The patient never takes his medication | -2 -1 0 +1 +2 |
| 14 | A brainstem stroke | Equal pupils | -2 -1 0 +1 +2 |
| 15 | A residual sedation | Moderate renal insufficiency | -2 -1 0 +1 +2 |
