# Supplementary material for: Pedagogical impact of different levels of e-learning teaching during anesthesia residency: a randomized clinical trial
Source: BMC Med Educ. 2026 Apr 29;26:971. doi: 10.1186/s12909-026-09225-4 (PMC13267392; doi:10.1186/s12909-026-09225-4)
Supplement: Supplementary file 3 — Supplementary Material 3. Pearson’s correlation analysis. [file 12909_2026_9225_MOESM3_ESM.docx]

**Supplementary File 3: Pearson’s correlation analysis**

|  | **Clinical Case/SCT pre-test**  (n=54) | **Clinical Case/SCT post-test**  (n=54) |
| --- | --- | --- |
| Pearson’s correlation | 0.41 | 0.31 |
| [95%IC]  P | [0.16; 0.61]  0.0021 | [0.04; 0.53]  0.0236 |

***Abbreviations****: SCT, script concordance test*
